# N-acylhydrazone inhibitors of influenza virus PA endonuclease with versatile metal binding modes

**DOI:** 10.1038/srep31500

**Published:** 2016-08-11

**Authors:** Mauro Carcelli, Dominga Rogolino, Anna Gatti, Laura De Luca, Mario Sechi, Gyanendra Kumar, Stephen W. White, Annelies Stevaert, Lieve Naesens

**Affiliations:** 1 Dipartimento di Chimica and Consorzio Interuniversitario di Ricerca in Chimica dei Metalli nei Sistemi Biologici, Università di Parma, Parco Area delle Scienze 17/A, 43124 Parma, Italy; 2 Dipartimento di Scienze del Farmaco e Prodotti per la Salute, Università di Messina, Polo Universitario SS. Annunziata, 98158 Messina, Italy; 3 Dipartimento di Chimica e Farmacia, Università di Sassari, Via Vienna 2, I-07100 Sassari, Italy; 4 Dept. of Structural Biology, St. Jude Children’s Research Hospital, 262, Danny Thomas Place, Mail Stop 311, Memphis, TN 38105 USA; 5 Rega Institute for Medical Research, KU Leuven – University of Leuven, B-3000 Leuven, Belgium

## Abstract

Influenza virus PA endonuclease has recently emerged as an attractive target for the development of novel antiviral therapeutics. This is an enzyme with divalent metal ion(s) (Mg^2+^ or Mn^2+^) in its catalytic site: chelation of these metal cofactors is an attractive strategy to inhibit enzymatic activity. Here we report the activity of a series of N-acylhydrazones in an enzymatic assay with PA-Nter endonuclease, as well as in cell-based influenza vRNP reconstitution and virus yield assays. Several N-acylhydrazones were found to have promising anti-influenza activity in the low micromolar concentration range and good selectivity. Computational docking studies are carried on to investigate the key features that determine inhibition of the endonuclease enzyme by N-acylhydrazones. Moreover, we here describe the crystal structure of PA-Nter in complex with one of the most active inhibitors, revealing its interactions within the protein’s active site.

Influenza virus is an enveloped virus with a segmented negative-oriented single-stranded RNA genome, belonging to the *Orthomyxoviridae*^1^. Seasonal influenza A and B viruses affect each year approximately 5–10% of the adult and 20–30% of the paediatric population^2^, and there is a permanent risk of sudden influenza pandemics, such as the notorious ‘Spanish flu’ in 1918^3^ and the swine-origin H1N1 pandemic in 2009^4^. Two classes of anti-influenza virus drugs are available, acting on the viral M2 ion-channel (amantadine and rimantadine) or on the viral neuraminidase (zanamivir and oseltamivir). The M2 inhibitors have limited clinical utility due to their central nervous system side effects^5^ and widespread resistance, as in the case of the 2009 pandemic H1N1 virus^6^,^7^,^8^; resistance is also a growing concern for oseltamivir^9^. Therefore, there is an urgent need for new antiviral drugs with an entirely different mode of action^10^.

The influenza virus polymerase complex is composed of three subunits: PB1, PB2 and PA^11^,^12^. The PA subunit performs the ‘cap-snatching’ endonuclease reaction, the PB2 subunit is responsible for initial binding of the capped RNAs, while the actual RNA synthesis is performed by the PB1 protein.

Given its crucial role in the viral life cycle, the influenza virus polymerase is widely recognized as a superior target for antiviral drug development^13^ and, in particular, inhibition of the PA endonuclease has deserved much attention in recent years^14^.

The endonuclease catalytic site resides in the N-terminal domain of PA (PA-Nter; residues 1~195)^15^,^16^,^17^. It comprises a histidine (His41) and a cluster of three strictly conserved acidic residues (Glu80, Asp108, Glu119), which coordinate (together with Ile120) one^18^, two^15^,^19^, or three^20^ manganese or magnesium ions. The two-metal-ion model is consistent with numerous biochemical findings^21^. Since the intracellular concentration of Mg^2+^ is at least 1000-fold higher than that of Mn^2+^, magnesium may be more biologically relevant^22^,^23^. A controversy about number and type of metal ions exists also for the active site of HIV-1 integrase^14^. HIV-1 integrase inhibitors are a paradigm for the innovative drug concept that is based on coordination with the metal cofactor(s) of viral enzymes^14^,^24^,^25^: similarly, several PA-binding agents with metal-chelating properties have been identified as influenza endonuclease inhibitors (Fig. 1), including 2,4-dioxobutanoic acid derivatives^26^,^27^, flutimide and its derivatives^28^, 2-hydroxyphenyl amide derivatives^29^, as well as tetramic acids^30^, 5-hydroxypyrimidin-4-one derivatives^31^, marchantins^32^ and green tea catechins, like epigallocatechin-3-gallate (EGCG, Fig. 1)^33^,^34^.

In recent years, we focused our research on chemical scaffolds that are able to chelate metal ions of PA-Nter, resulting in inhibition of influenza virus replication^29^,^35^. N-acylhydrazones represent an appealing class of chelating ligands with a broad spectrum of biological activities^36^,^37^, such as activity against HIV^38^,^39^,^40^, hepatitis A, vaccinia^41^,^42^ and influenza virus^43^. In the present work, we report the biological activity of a series of N-acylhydrazones (Fig. 2), as determined in an enzymatic assay with PA-Nter endonuclease as well as in cell-based influenza viral ribonucleoprotein (vRNP) reconstitution and virus yield assays. Several N-acylhydrazones were found to have promising anti-influenza activity with 50% effective concentration values (EC_50_) in the range of 3–20 μM and good selectivity (Table 1 and Fig. 3). Computational docking studies of two candidate ligands in the PA-Nter active site gave information about the features that could determine inhibition of endonuclease activity. Moreover, we describe the X-ray crystal structure of PA-Nter in complex with one of the most active inhibitors.

## Results and Discussion

### Chemistry

N-acylhydrazones **1–27** (Fig. 2) were prepared in high yields by following literature methods^42^ (Fig. 2A); they were characterized by spectroscopic tools, mass spectrometry and elemental analysis. Even if isomerism around the C = N bond is possible, **1–27** are present in the *E* form in solution, as evidenced by the chemical shift values of the *H*C = N and N*H* protons in the ^1^H-NMR spectrum^44^. Exceptions are represented by the alkyl-derivatives **3** and **4** (2:1 and 5:3 *E:Z ratio*, respectively).

If R’ (Fig. 2A) is a 2-hydroxy substituted phenyl ring, the corresponding acylhydrazones can coordinate one or, depending on denticity, two metal centers (modes A and B in Fig. 4). Starting from N’-(2,3-dihydroxybenzylidene)-semicarbazide (**1**) and its methoxy-analogue (**2**), we modified the acylhydrazonic substituent R” (**3**–**8**, **18**, **19**, Fig. 2A). In **18** and **19**, also the gallic moiety can be involved in the chelation of the metal cofactors (mode C, Fig. 4). In order to investigate the role of hydroxyl substituents **9–11**, **13–17**, **20–23** and **27** were also synthesized. Compound **12** was synthesized in order to confirm the crucial influence of the gallic moiety. Finally, **26** was here considered, because it is an inhibitor of HIV RNase H^38^, another enzyme with two magnesium ions in its active site.

Since the inhibitory activity of the N-acylhydrazones could be related to chelation of the divalent metal cofactor(s) in the influenza PA-Nter active site, we investigated the coordination properties of one model ligand (i.e. **19**, **H**_**2**_**L**) towards Mg^2+^. Different reaction conditions were used (1:1 and 1:2 metal to ligand *ratio*, up to 4 equivalents of triethylamine), but in any case the same chemical species **Mg(HL)**_**2**_**∙4H**_**2**_**O** was recovered and conveniently characterized. The use of a coordinating solvent as d_6_-DMSO causes partial decoordination of the ligand, but the ^1^H-NMR spectrum in MeOD, instead, shows only the signals attributable to the complex. In the ^13^C-NMR spectrum, the signal of the C = O quaternary carbon is practically unaffected by complexation, suggesting that the C = O group is weakly involved in the coordination to the metal ion. This is confirmed, in the IR spectrum, by the shift of about 20 cm^−1^ of the C = O absorption, while a shift of 30–50 cm^−1^ is expected when the carbonylic oxygen is tightly bound to the metal ion^42^. ESI-mass spectra and elemental analysis confirmed the formula **Mg(HL)**_**2**_**∙4H**_**2**_**O**.

The interaction between the N-acylhydrazone ligands and the magnesium cation was investigated also by means of UV-visible spectroscopy (UV-visible titrations of **23** and **19** with increasing amount of Mg(CH_3_COO)_2_ are shown in Figure S1). The spectrum of **19** includes a band at 313 nm assignable to n-π^*^ transitions of the C = N and C = O groups. By adding increasing equivalents of Mg(CH_3_COO)_2_, the absorption around 400 nm increases, and a new band appears with a maximum at 397 nm. The opposite trend is observed in the range 300–350 nm, where an isosbestic point is present close to 335 nm. When the same experiment was performed with **23**, a different behavior was observed. Increasing concentration of Mg^2+^, in fact, caused a diminution in the maximum absorption, an isosbestic point is visible at about 345 nm, but a new band at 400 nm does not appear. Ligands **19** and **23** coordinate the Mg^2+^ ions in different ways: **19** chelates the metal ion by using the deprotonated salicyl oxygen and the iminic nitrogen, while for **23**, the gallic moiety is supposed to be involved (Fig. 4A,B *versus* C), leading to different, less extensive, modifications of the UV spectrum. These results will be revisited during the discussion of the biological activity.

### Inhibition of the PA-Nter enzyme

All the compounds were tested for their ability to inhibit the influenza endonuclease in an enzymatic plasmid-based assay with recombinant PA-Nter^27^, as well as in cell-based influenza methods (i.e. virus yield and vRNP reconstitution assays)^45^. The results are shown in Table 1 and summarized in Fig. 3 to visualize the structure-activity relationships; Figure S2 shows the dose-response curves for three representative compounds (i.e. **10**, **13** and **23**) in either the PA-enzyme or vRNP reconstitution assay.

The moderate activity (IC_50_ = 24 μM) of N’-2,3-dihydroxybenzylidene semicarbazide (**1**) was completely lost when the NH_2_ moiety was replaced by a hydrophobic heptyl chain (**3**), but it is less affected when a phenyl or a 2-hydroxyphenyl is present (**5** and **7**, IC_50_ = 84 and 54 μM, respectively). When the hydroxyl in position 3 on R1 (2,3-dihydroxybenzylidene) was replaced by a methoxy group (2-hydroxy-3-methoxybenzylidene), the activity disappeared (compounds **2**, **4**, **6** and **8**). The activity is unaffected (IC_50_ values ranging from 45 to 75 μM) when going from two hydroxyls in R1 (**7**) to compounds with three hydroxyls (i.e. **9, 10** and **11**). Similarly, **11** (R1 = 3,4,5-trihydroxyphenyl, R2 = 2-hydroxyphenyl) had comparable activity as **27** (R1 = 3,4,5-trihydroxyphenyl, R2 = NH_2_). Within the series carrying a 2-hydroxyphenyl R2 group, the activity of **11** is particularly intriguing. **11** does not have the possibility to chelate in a tridentate ONO fashion (mode A in Fig. 4), but it can coordinate two cations by means of its three OH groups in R1 (mode C, Fig. 4). Note that a similar chelating mode was observed in a crystal structure, solved by Cusack and coworkers^46^, of PA-Nter endonuclease in complex with the inhibitor EGCG.

The PA-Nter inhibitory activity strongly depends on the number and position of hydroxyl substituents in R1 and R2: this is clearly highlighted by the data obtained with compounds **13–23**, in which R2 is a 3,4,5-trihydroxyphenyl (gallic) group, the most active scaffold in our series. The analogue carrying an unsubstituted aromatic ring as R1 (compound **13**) had moderate activity (IC_50_ = 69 μM). When one OH was added at position 2 of the R1 ring (**14**), the activity was lost. Adding a second OH substituent at position 5 resulted in strong activity (compound **15**, IC_50_ = 9 μM); medium activity for a 3-OH (**18**; IC_50_ = 83 μM), and marginal activity when the second OH is at position 4 (**17**, IC_50_ ≥ 370 μM). The addition of a 3-methoxy group (**19**) abolished all inhibitory activity. This cannot be related to variations in the chelating features displayed by the R1 moiety, since compounds **14–19** all have, in theory, the capacity to chelate one metal ion through the ortho-OH and iminic nitrogen (mode A in Fig. 4). Moreover, compound **18** can, in principle, chelate the two M^2+^ ions in the active site according to mode B (Fig. 4), yet it (IC_50_ = 83 μM) has nine-fold lower activity than **15**, that does not possess this two-metal chelating feature. Therefore, we hypothesized that the inhibitory activity of the series containing the gallic moiety is determined by: (i) the capacity of the moiety R2 to chelate two metal ions in the active site of the enzyme, according to mode C (Fig. 4); and (ii) the presence and position of one or more hydroxyl substituents in R1, which may possibly result in ligand-protein interactions (e.g. through hydrogen bonds). This assumption was supported by molecular docking calculations and X-ray analysis of inhibitor **23** in complex with PA-Nter (*vide infra*). At this point, change of the substituents in R1 represents the next logical step. Substitution of the 5-hydroxyl in **15** by a methoxy group (**16**) causes a dramatic drop in activity (IC_50_ = 9 and 454 μM for **15** and **16**, respectively). When two or three OH groups are present in R1, their spatial disposition greatly affects the activity. In particular, all the compounds with a trihydroxylated phenyl group as R1 (i.e. **20**, **21**, **22** and **23**) were able to inhibit PA-Nter quite potently. The lowest IC_50_ values were obtained for **21** and **23** (IC_50_ = 13 and 7 μM, respectively), which both have one of their three hydroxyl groups at position 5. The most active compound in this series was **23**, which lacks the hydroxyl group at position 2 of R1, further confirming that this function is undesirable or even detrimental for inhibitory activity against PA-Nter, as already noticed above for **14**.

Consistent with a crucial role of the R2 gallic moiety in metal chelation, the strong activity of **15** was completely lost in its 3,4,5-trimethoxy analogue **12**. On the other hand, the R2 gallic containing compounds displayed moderate activity (IC_50_ values around 40 μM) when R1 was absent (i.e. the 3,4,5-trihydroxybenzohydrazide **28**, Fig. 2), or composed of an extended ring system (**26**) or a pyrrole ring (**25**). Still lower activity was seen with the pyridine analogue **24**. Evidently, the 3,4,5-trihydroxybenzyl moiety at R2 is fundamental but not sufficient to ensure potent PA-Nter endonuclease inhibition, since the interactions of R1 with the amino acid side chains of the protein appear crucial in modulating activity.

### Inhibition of vRNP activity or virus replication in cells

To determine the anti-influenza virus activity of compounds **1**–**28** in cell culture, we performed an influenza vRNP reconstitution assay in human embryonic kidney 293 T (HEK293T) cells, then subjected the active compounds (i.e. EC_50_ < 100 μM) to a virus yield assay in influenza virus-infected Madin-Darby canine kidney (MDCK) cells (Table 1 and Fig. 3). For some N-acylhydrazone compounds, we observed quite potent and selective activity in the vRNP reconstitution assay. This indicates that they are able to inhibit viral RNA synthesis and suggests that they could be classified as original PA inhibitors. Values for EC_50_ (vRNP) or EC_90_ (virus yield) in the range of 0.4–18 μM were obtained for compounds **15** and **20–23**, which all carry a 3,4,5-trihydroxyphenyl as R2, and possess either two (**15**) or three (**20–23**) hydroxyl substituents in the R1 moiety. As in the enzymatic PA-Nter assays, the compounds having R2 as a gallic moiety (Fig. 3: **21**, **22** and **23**) showed slightly higher activity than the compounds carrying a 2-hydroxyl R2 group (**9**, **10** and **11**); **10** and **22** have substantially the same EC_50_ in the vRNP reconstitution assay in HEK293T cells.

The hydrazide **28** displayed weak (virus yield) to moderate (vRNP reconstitution) activity, albeit less than the most active molecules in the 3,4,5-trihydroxyphenyl series (i.e. **18** and **21–23**). Even if there are no data indicating that the compounds reported in the paper are subject to hydrolysis, the activity of **28** could raise the concern that for some N-acylhydrazones the antiviral activity in cell culture may be related to their intracellular hydrolysis. However, this is unlikely, since the antiviral potency showed large differences (i.e. EC_50_ values between 0.42 and 29 μM) for compounds with the same R2 but different R1 groups, meaning that R1 does play a role in modulating the antiviral effect.

Most compounds carrying as R1 a 2,3-dihydroxybenzylidene (i.e. **3**, **5** and **7**) or 2-hydroxy-3-methoxybenzylidene moiety (i.e. **4**, **6** and **8**) showed relatively high cytotoxicity in the vRNP assay, with CC_50_ values below 50 μM and a selectivity index (ratio of CC_50_ to EC_50_) below 8. Two notable exceptions are **18** and **19** (containing a 2,3-dihydroxybenzylidene or 2-hydroxy-3-methoxybenzylidene R1, respectively) which were not cytotoxic at 200 μM and displayed favorable antiviral selectivity.

Some N-acylhydrazone compounds were devoid of activity in the enzymatic assay, yet showed good to moderate efficacy in cell culture (e.g. **14** and **19**, having EC_50_ values of 2.2 and 7.1 μM, respectively). For most of the active compounds (i.e. **9**, **11**, **13**, **15–21**, **23**, **24** and **26**) a fair correlation was seen for the two cell-based assays, since the EC_50_ values obtained in the vRNP assay were maximum 5-fold different from the EC_90_ values in the virus yield assay. On the other hand, this difference was 8-fold or more for **7**, **10**, **14**, **22**, **25** and **28**. Some N-acylhydrazone compounds showed good to moderate efficacy in the vRNP assay (e.g. **14** and **19**, having EC_50_ values of 2.3 and 5.7 μM, respectively), yet were devoid of activity in the enzymatic assay. This observation suggests that they may inhibit the viral polymerase in an endonuclease-independent manner. To achieve a clear insight into the antiviral profile of the N-acylhydrazones, specific mechanistic experiments are currently ongoing in our laboratory, in which we are analyzing in full depth their effects on virus entry, polymerase-dependent RNA synthesis or the late stage (maturation and release) of the virus replication cycle.

### Docking studies

In order to explore the possible binding mode of the synthesized compounds, docking simulations by GOLD program^47^ were performed by using the structural coordinates (PDB code 4AWM) for the PA-Nter endonuclease in complex with EGCG^46^. Considering that the position of the side-chains of some residues changes depending on which pocket the ligand is occupying, we superimposed some X-ray structures of complexes between PA-Nter endonuclease and known active ligands. It was observed that the side-chain of amino acid Tyr24 shows greater movement than the other residues and for this reason we considered it as a flexible residue during the docking procedure.

**Figure 1:**
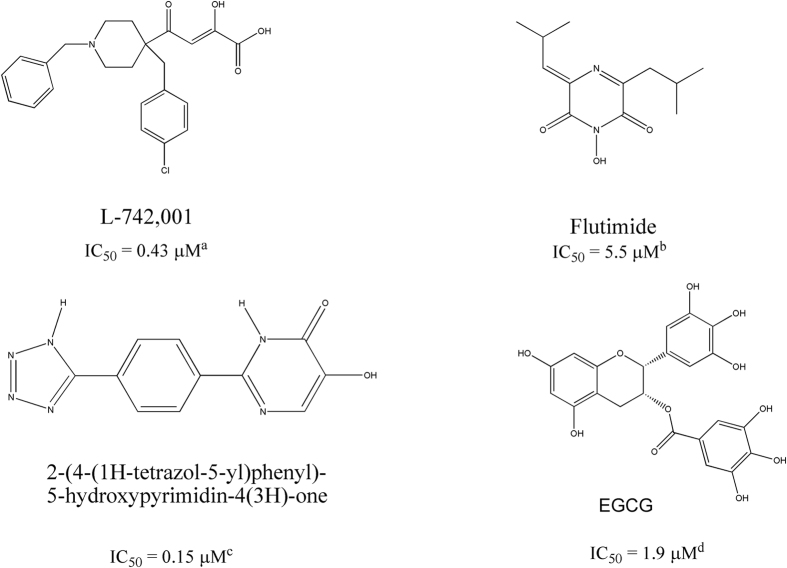
Chemical structures of some prototype inhibitors of influenza virus endonuclease. Inhibitor activity in enzymatic assays (IC_50_, μM) as reported in: ^a^ref. 25, ^b^ref. 27, ^c^ref. 30, ^d^ref. 49.

**Figure 2:**
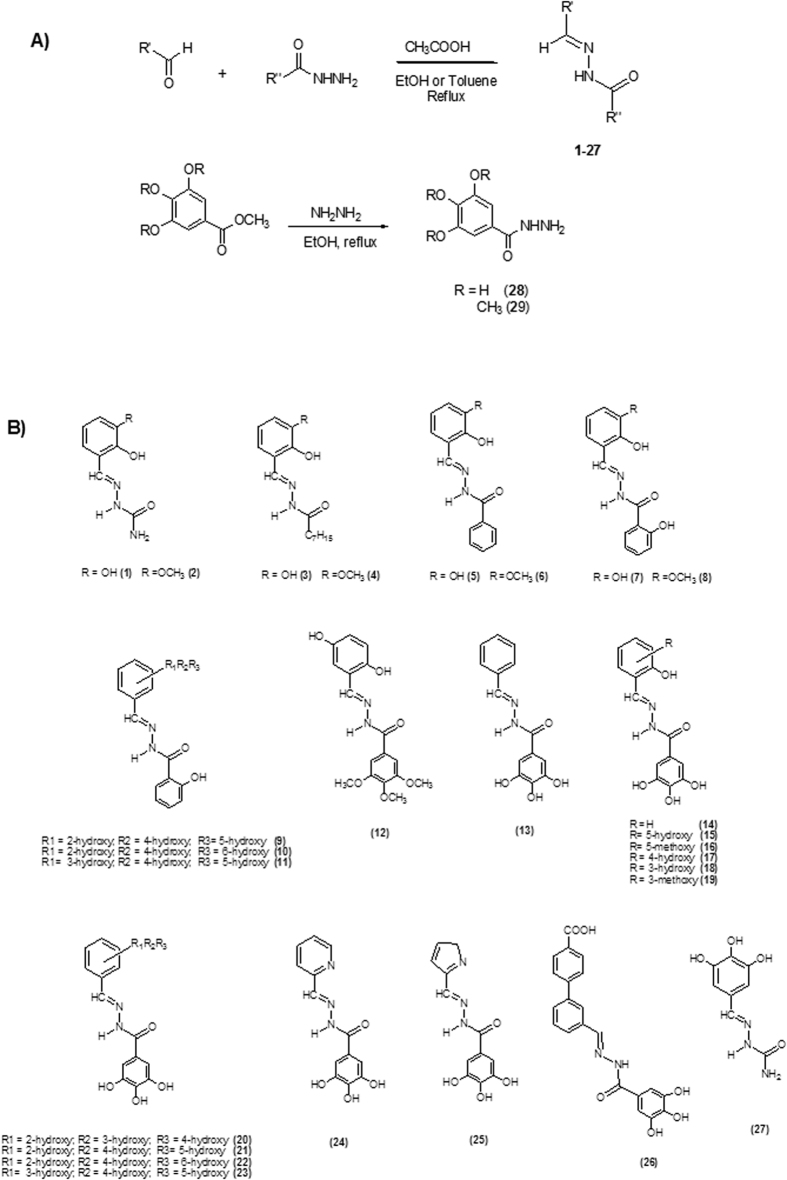
General synthesis for N-acylhydrazones 1–27 and hydrazides 28 and 29 (A). Chemical structures of compounds 1–27 (B).

**Figure 3:**
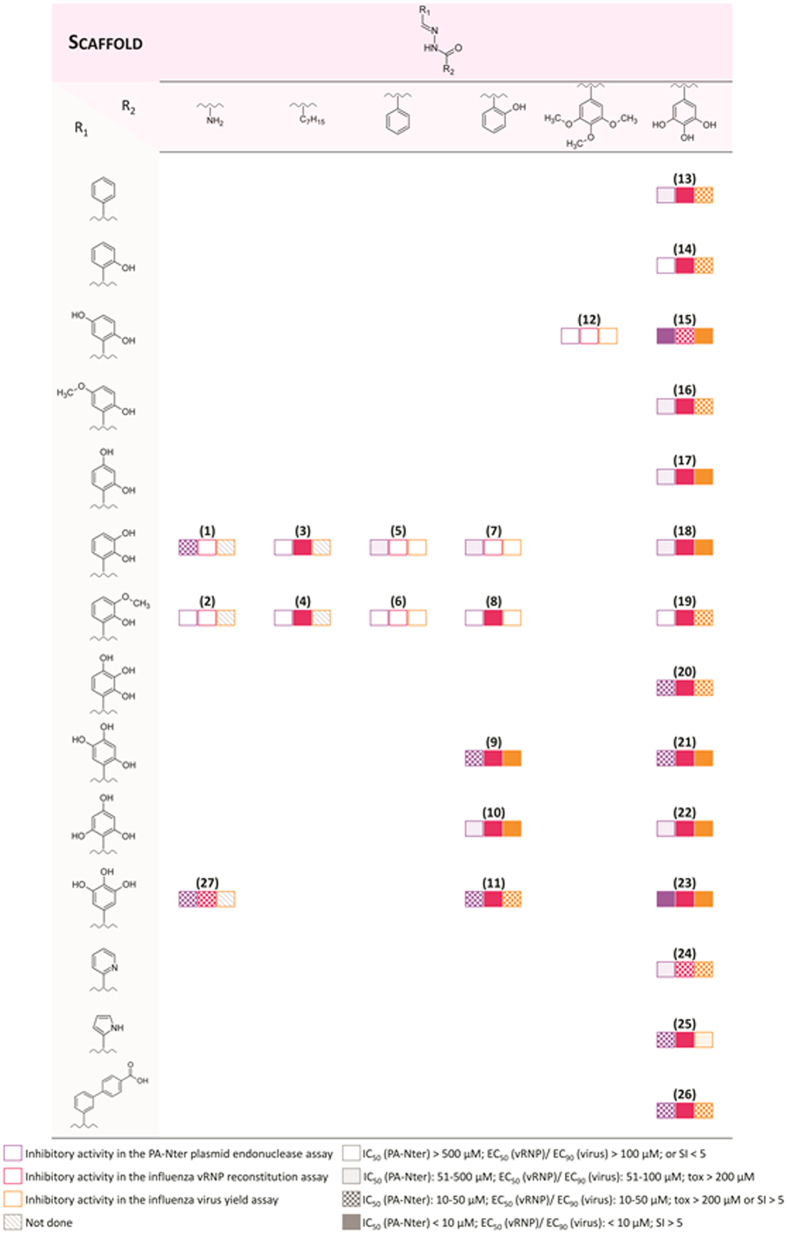
Overview of the structure-activity relationship for compound**s** 1–27.

First, test docking calculations, using EGCG, L-742,001 and 2-(4-(1*H*-tetrazol-5-yl)phenyl)-5-hydroxypyrimidin-4(3*H*)-one (Fig. 1), were carried out to compare experimental and predicted binding modes and validate docking procedure. Their best docking poses agreed well with the experimental binding modes (rmsd values of 0.8, 1.2 and 0.7, respectively).

**Figure 4:**
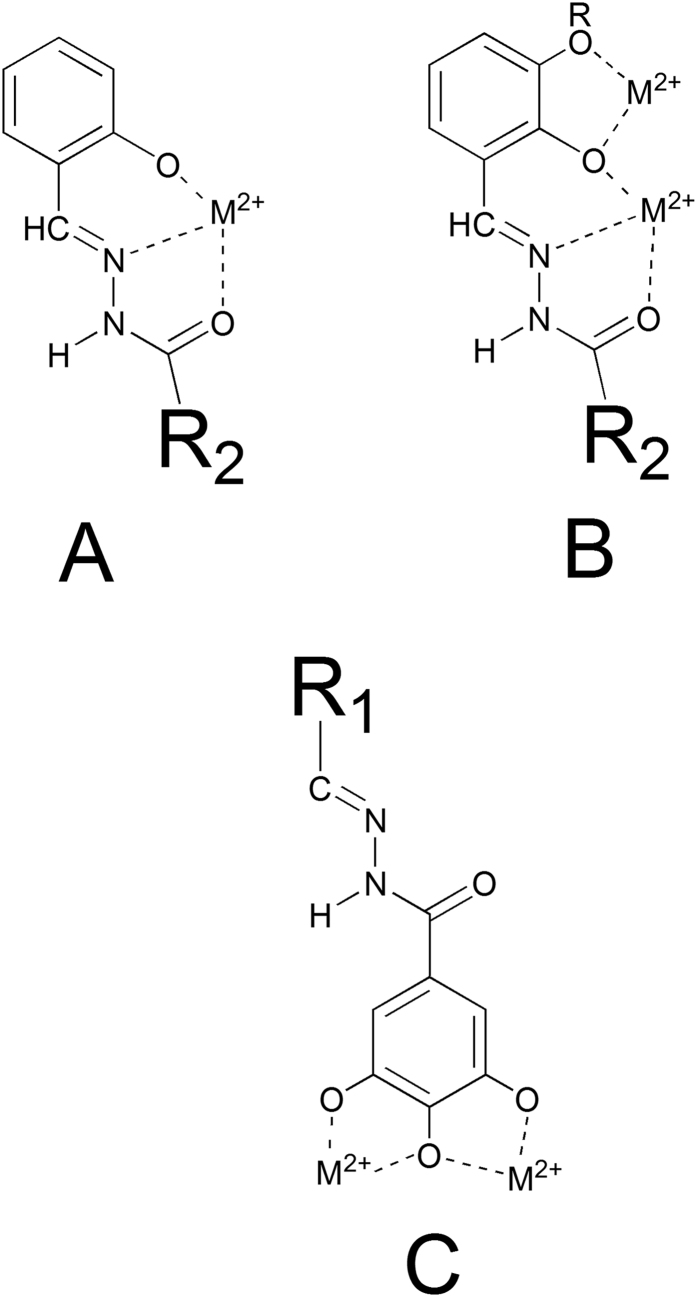
Scheme of possible binding modes of the studied N-acylhydrazones.

Next, docking of several N-acylhydrazones was performed and this generated a number of possible binding conformations, highlighting that the active site cavity of the PA endonuclease is quite spacious, as already demonstrated by crystallographic studies^19^,^46^, and confirming the ability of this scaffold to chelate the two M^2+^ ions in different ways (Mode A-C in Fig. 4).

**Figure 5:**
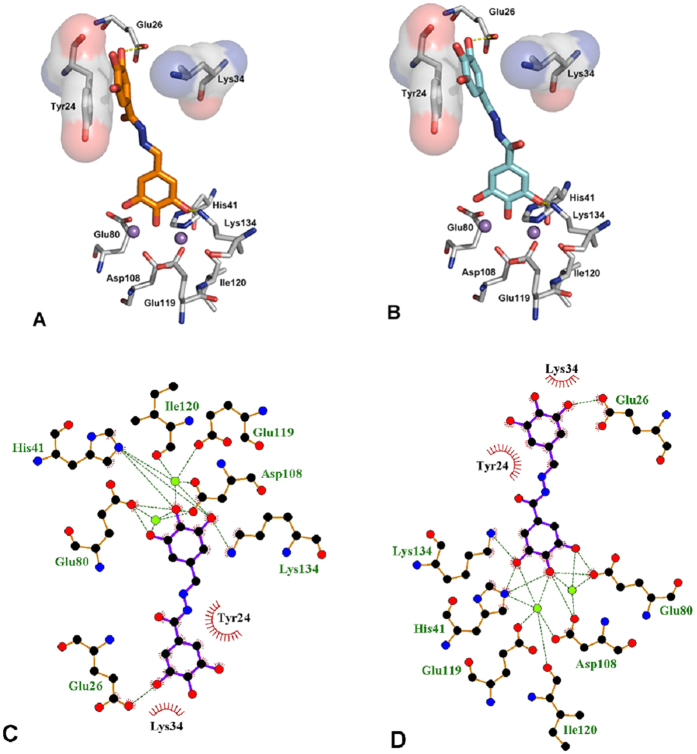
First (**A**) and second (**B**) GOLD cluster docked solutions of compound **23** (orange and cyan, respectively) in complex with PA endonuclease. Key residues of the pocket are presented using PyMOL [ http://www.pymol.org] and LIGPLUS [Laskowski, R. A.; Swindells, M. B. *Journal of chemical information and modeling* **2011,** *51*, 2778]. Hydrogen bonds are illustrated by dotted lines, while the divalent metal ions are shown as purple spheres. Schematic drawings of the interactions of the first (**C**) and second (**D**) GOLD cluster docked solutions generated using LIGPLUS. Dashed lines are hydrogen bonds and ‘eyelashes’ show residues involved in hydrophobic interactions.

Figure 5 displays the first (panel A) and second (panel B) GOLD cluster docked solutions for compound **23.** These two complex structures represent the largest clusters with similar fitness values (59.20 and 58.65, respectively). In both cases, **23** appears able to coordinate the two M^2+^ ions in the active site through the three contiguous OH groups (Fig. 5). In addition, **23** was predicted to form two hydrogen bonding interactions, i.e. with the catalytic Lys134^48^ on the one side and Glu26 on the other side. Furthermore, in these two different binding modes, **23** forms π–π interactions with the aromatic ring of Tyr24, in a fashion similar to that described for other endonuclease inhibitors, i.e. EGCG^46^ and L-742,001^19^.

**Figure 6:**
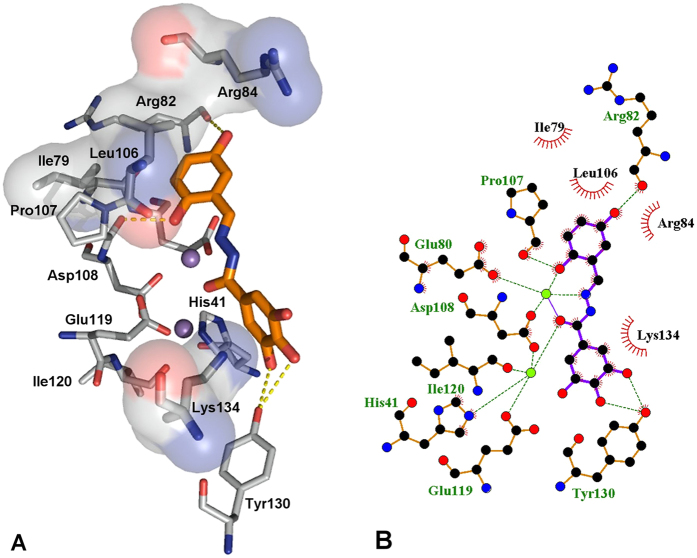
(**A**) Binding mode of compound **15** (orange) in complex with PA endonuclease. Key residues of the pocket are presented using PyMOL [ http://www.pymol.org] and LIGPLUS [Laskowski, R. A.; Swindells, M. B. *Journal of chemical information and modeling* **2011,** *51*, 2778]. Hydrogen bonds are illustrated by dotted lines while the divalent metal ions are shown as purple spheres. (**B**) Schematic drawing of the interactions of compound **15** generated using LIGPLUS. Dashed lines are hydrogen bonds and ‘eyelashes’ show residues involved in hydrophobic interactions.

The best docked conformation for compound **15** (Fig. 6, fitness value 68.56), which has an activity slightly lower than **23,** reveals a different role for the gallic moiety. The ligand seems to form two hydrogen bonding interactions with Tyr130 as well as a cation–π interaction with Lys134. Tyr130 lies in a pocket that also contains Arg124, a residue that was proposed to have a crucial role in binding of the RNA substrate^16^,^45^,^49^. Compound **15** appears further stabilized by hydrogen bonding interactions between two hydroxyl groups and Arg82 and Asp108. In this case, chelation of the two M^2+^ ions is carried out by involving the imine group (mode A in Fig. 4).

It is important to highlight that compounds **23** and **15**, although in different ways, both are able to chelate the metal cofactors and to establish interactions with highly conserved aminoacids (Tyr24, Glu26, Arg124, Tyr130 and Lys134) that are very important for both endonuclease activity and transcription *in vitro*^30^,^46^,^50^. The crucial role of such interactions is underlined by the differences in activity between **15** (IC_50_ = 9.0 μM) and **19** (>500 μM): their coordinating features are similar, since both coordinate to the divalent metal ion through the phenolic oxygen, the iminic nitrogen and the carbonylic oxygen (mode A in Fig. 4), but the biological activity could be related to their different ability to engage interactions with the protein environment.

### Crystallographic Studies

**Figure 7:**
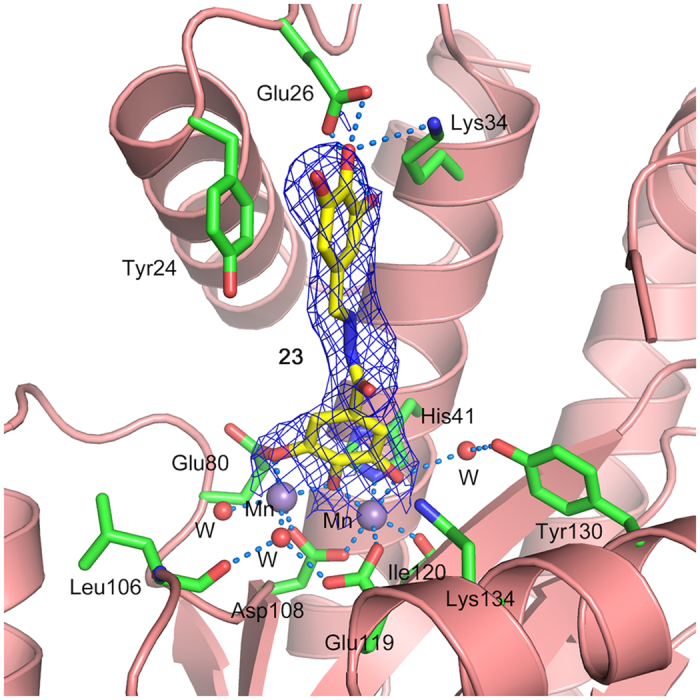
Crystal structure of PA_N_^ΔLoop^ in complex with compound 23. Active site residues are shown in sticks with green carbons, manganese atoms are shown as purple spheres and water molecules as red spheres. Compound **23** is shown in sticks with yellow carbons. Protein secondary structure is shown as ribbons in salmon color. *2Fo-Fc* electron density map contoured at 1σ is shown as blue mesh. Hydrogen bonds and metal coordination are shown with dotted lines. The H-bond distances from the side chain carboxyl group of Glu26 to *p*-OH and *m*-OH of the trihydroxyphenyl group of the inhibitor are 2.7 Å and 3.0 Å, respectively. The H-bond distance from the side chain of Lys34 to *p*-OH of the trihydroxyphenyl group is 3.6 Å. The H-bond distance to the water molecule from *m*-OH of the galloyl moiety is 3.0 Å, which in turn is H-bonded to the side chain of Tyr130 with a distance of 2.7 Å. Crystal structure has been deposited in the RCSB Protein Data Bank with PDB ID: 5EGA.

Attempts were made to co-crystallize PA-Nter with **15**, **20**, **21** and **23** in one to four molar excess. While crystals appeared and diffracted well, upon data processing, no or very little electron density for the inhibitors was observed. Attempts to soak apo crystals in crystallization solution containing 5 mM inhibitor overnight also did not result in substantial electron density for the inhibitor. As a last resort, dry powder of the inhibitor was sprinkled over the crystallization drop containing apo crystals and left over night. This experiment was successful for compound **23**, the crystals diffracted to 2.15 Å and diffraction data were collected (PDB ID 5EGA). The refined structure shows unambiguous electron density for the inhibitor (Table S1 and Fig. 7). The complex structure confirms one of the two binding modes predicted by the docking simulations (Fig. 5, panel B). The galloyl moiety chelates the manganese ions, while the trihydroxyphenyl group stacks against the Tyr24 side chain. It is interesting to note that two of these hydroxyl groups are in position to form hydrogen bonds with the side chain of Glu26 and Lys34 (Fig. 7). These interactions suggest that other functional groups, e.g. halogens, could be used in place of the hydroxyl groups for better interactions with Glu26 and Lys34 side chains, and the inhibitory potency of these compounds could be further improved.

## Conclusions

The development of new agents for the treatment of influenza infection that exert their action by inhibition of the endonuclease activity of influenza RNA-dependent RNA polymerase is a strategy that recently is gaining a lot of interest^13^,^27^,^31^,^43^,^51^,^52^. The results here presented add the N-acylhydrazone scaffold to the library of the chelating molecules with potent antiviral activity (EC_90_ < 5 μM, virus yield assay in influenza virus-infected MDCK cells). The structure of the N-acylhydrazone **23** co-crystallized with PA-Nter is important not only because confirms that the polyhydroxypheyl group efficiently coordinates two metal ions in the active site of the enzyme^46^, but also because highlights the importance of the (flexible) inhibitor backbone in order to engage effective interactions with crucial aminoacids of the protein. Inhibition of the endonuclease activity of influenza RNA-dependent RNA polymerase could represent another example, after carbonic anhydrase, histone deacetylase, and HIV-1 integrase, of metal binding as a successful strategy in drug design^14^.

## Experimental Section

### Materials and methods. Chemistry

All reagents of commercial quality were purchased from Sigma-Aldrich and used without further purification. The purity of the compounds was determined by elemental analysis and verified to be ≥95% for all synthesized molecules. NMR spectra were recorded at 25 °C on a Bruker Avance 400 FT spectrophotometer. The attenuate total reflectance IR spectra were recorded by means of a Nicolet-Nexus (Thermo Fisher) spectrophotometer by using a diamond crystal plate in the range of 4000–400 cm^−1^. Elemental analyses were performed by using a FlashEA 1112 series CHNS/O analyzer (Thermo Fisher) with gas-chromatographic separation. Electrospray mass spectral analyses (ESI-MS) were performed with an electrospray ionization (ESI) time-of-flight Micromass 4LCZ spectrometer. MS spectra were acquired in positive EI mode by means of a direct exposure probe mounting on the tip of a Re-filament with a DSQII Thermo Fisher apparatus, equipped with a single quadrupole analyzer. UV–Vis spectra were recorded on an Evolution 260 Bio Thermo spectrophotometer by using cells of 1 cm path length. UV-vis absorption spectra of **19** and **23** were registered using a ca. 10^−5^ M solution in methanol. Each metal/ligand system was studied by titrating a 2.8 ml sample of the ligand solution with a methanolic solution of Mg(CH_3_COO)_2_; 8–12 spectra of samples with M:L molar *ratio* ranging from 0 to 6 were measured.

### Synthesis of the ligands (general procedure)

All the N-acylhydrazones were prepared in a manner similar to reported procedures^42^. Briefly, to a solution of the aldehyde in absolute ethanol or toluene, an equimolar amount of the hydrazide dissolved in the same solvent was added. The mixture was refluxed for 6 hours, cooled at room temperature and concentrated in vacuum. The resulting precipitate was filtered off, washed with cold ethanol and dried in vacuum.

3,4,5-trihydroxybenzohydrazide (**28**) and 3,4,5-trimethoxybenzohydrazide (**29**) were obtained by reaction of the corresponding methyl esters with hydrazine monohydrate^53^. Hydrazine was added to an ethanol suspension of the ester and stirred at room temperature until the solute completely dissolved. Reaction mixture was then refluxed overnight. On concentrating the solution, a precipitate was observed, which was filtered and washed with cold ethanol. Chemical characterization of **1–29** and of **Mg(HL)**_**2**_ **4H**_**2**_**O** is collected in the Supplementary Information.

### Computational Studies

The crystal structure of PA-Nter endonuclease in complex with EGCG was retrieved from the RCSB Protein Data Bank (entry code 4AWM). The ligand and water molecules were discarded and the hydrogens were added to the protein by Discovery Studio 2.5. The charge on the metal ions was set as +2. EGCG, L-742,001, and 2-(4-(1*H*-tetrazol-5-yl)phenyl)-5-hydroxypyrimidin-4(3*H*)-one structures were extracted from their X-ray complexes (PDB IDs 4AWM, 4W9S and 4E5H respectively). The other ligand structures were constructed using Discovery Studio 2.5.5 (Accelrys, Discovery Studio) and energy minimized using the Smart Minimizer protocol (1000 steps) which combines the Steepest Descent and the Conjugate Gradient methods.

The minimized ligands were docked in their corresponding proteins by means of GOLD Suite 5.0.1^54^. The region of interest used by the GOLD program was defined in order to contain the residues within 15 Å from the original position of the ligand in the X-ray structure. The side-chain of residue Tyr24 was allowed to rotate according to the internal rotamer libraries in GOLD Suite 5.0.1. GoldScore was chosen as fitness function. The standard default settings were used in all calculations and the ligands were submitted to 100 genetic algorithm runs. The “allow early termination” command was deactivated. Results differing by less than 0.75 Å in ligand-all atom rmsd, were clustered together. The best GOLD-calculated conformation was used both for analysis and representation.

### Plasmid-based endonuclease assay

This enzymatic assay was performed according to a previously published method^27^. One microgram of recombinant PA-Nter (residues 1–217 from the PA protein of influenza virus strain A/X-31) was incubated with 1 μg (16.7 nM) of single-stranded circular DNA plasmid M13mp18 (Bayou Biolabs, Metairie, Louisiana) in the presence of the test compounds and at a final volume of 25 μL. The assay buffer contained 50 mM Tris-HCl pH 8, 100 mM NaCl, 10 mM β-mercaptoethanol and 1 mM MnCl_2_. The reaction was incubated at 37 °C for 2 h and then stopped by heat inactivation (80 °C, 20 min), followed by visualization of the endonucleolytic digestion of the plasmid by gel electrophoresis on a 1% agarose gel with ethidium bromide staining. The amount of remaining intact plasmid was quantified by ImageQuant TL software (GE Healthcare, Diegem, Belgium). The percentage inhibition of PA endonuclease activity was plotted against the compound concentration on a semi-logarithmic plot, using GraphPad Prism software (GraphPad Software, La Jolla, CA). The 50% inhibitory concentrations (IC_50_) were obtained by nonlinear least-squares regression analysis of the results from three independent experiments. 2,4-Dioxo-4-phenylbutanoic acid (DPBA; Interchim, Montluçon, France) was included as the reference compound.

### Cells and media

MDCK cells (a kind gift from M. Matrosovich, Marburg, Germany) and HEK293T cells (purchased from Thermo Fisher Scientific, Waltham, MA) were cultivated in Dulbecco’s modified Eagle medium supplemented with 10% fetal calf serum, 1 mM sodium pyruvate, and 0.075% sodium bicarbonate. During virus experiments, the MDCK cells were maintained in MDCK infection medium, consisting of Ultra MDCK medium (Lonza, Basel, Switzerland) supplemented with 0.0225% sodium bicarbonate, 2 mM L-glutamine, and 2 μg/ml tosyl phenylalanyl chloromethyl ketone-treated trypsin (Sigma-Aldrich, St. Louis, MO). The cells were incubated in a humidified atmosphere containing 5% CO_2_.

### vRNP reconstitution assay

The procedure to determine the inhibitory effect of the compounds on influenza virus vRNPs reconstituted in HEK293T cells, is described in full detail elsewhere^45^. Briefly, the four relevant plasmids (i.e. the expression plasmids for PB1, PB2, PA and NP) were combined with the fluc reporter plasmid, and co-transfected into HEK293T cells using Lipofectamin 2000 (Invitrogen, Life Technologies, Gent, Belgium). After incubation at 37 °C for 24 h in the presence of serial dilutions of the test compounds, the ONE-Glo luciferase assay system (Promega, Madison, WI) was used to determine luciferase activity. EC_50_ was defined as the compound concentration causing 50% reduction in the vRNP-driven firefly luciferase signal, as compared to cells receiving medium instead of compound. These EC_50_ values were calculated by interpolation assuming a semi-log dose-response effect using GraphPad Prism software. In parallel, compound cytotoxic activity was determined in untransfected HEK293T cells which had been incubated with serial dilutions of the compounds for 24 h, using the 3-(4,5-dimethylthiazol-2-yl)-5-(3-carboxymethoxyphenyl)-2-(4-sulfophenyl)-2H-tetrazolium (MTS) cell viability assay (CellTiter 96 AQ_ueous_ One Solution Cell Proliferation Assay; Promega). These spectrophotometric data were used to calculate the 50% cytotoxic concentration (CC_50_), i.e. the concentration reducing cell viability by 50%, as compared to wells receiving medium instead of compound. Ribavirin (Virazole; ICN Pharmaceuticals, Costa Mesa, CA) was included as the reference compound.

### Virus yield assay

We previously published in full detail the virus yield assay to determine the anti-influenza virus activity in MDCK cell cultures^55^. Briefly, one day prior to infection, MDCK cells were seeded into 96-well plates at 25,000 cells per well. At day 0, serial dilutions of the test compounds were added, immediately followed by infection with influenza A/PR/8/34 virus. After 24 h incubation at 35 °C, the virus amount in the supernatants was estimated by determining the viral genome copy number in a one-step quantitative real-time reverse transcription polymerase chain reaction (qRT-PCR) assay (CellsDirect One-Step qRT-PCR kit; Invitrogen), with influenza virus M1-specific primers and probe. The compound concentration values causing a 2-log10 (EC_99_) and a 1-log_10_ (EC_90_) reduction in viral RNA (vRNA) copy number at 24 h p.i., as compared to the virus control receiving no compound, were calculated by interpolation from data of at least three experiments. In parallel, the CC_50_ values after 24 h incubation with compounds were determined in uninfected MDCK cells, using the spectrophotometric MTS cell viability assay described above, respectively. Ribavirin was included as the reference compound.

### Crystallographic analysis

A PA_N_ construct (PA_N_^ΔLoop^) with a loop (residues 51–72) deleted and replaced with GGS from A/California/04/2009 H1N1 strain was used for the crystallographic studies. The details of cloning, over-expression and purification are described elsewhere^19^,^56^. Briefly, the gene was cloned into pET52b vector and transformed into BL21 (DE3) cells, and the protein was expressed in LB medium overnight at 18 **°**C after induction at an OD_600_ ~0.8 with 0.2 mM isopropyl-β**-**thiogalactopyranoside (IPTG). The protein was purified from cell lysates by HisTrap affinity chromatography and the 10xHis tag was removed by digestion with thrombin. The protein was further purified by gel filtration using a Superdex 75 size-exclusion chromatography column in 20 mM Tris pH 8.0, 150 mM NaCl and 1 mM TCEP. The protein was concentrated to 10–12 mg/ml for crystallization. Crystals were grown in 0.2 M MgCl_2_, 2 mM MnCl_2_, 0.1 M Tris pH 8.5, 30% (w/v) PEG 4000 using the hanging drop method. For determination of the protein-inhibitor complex structure, the powder of the inhibitor was sprinkled on a 2 μl drop of a 1:1 ratio mixture of protein solution and well solution, on a cover slide hanging over 500 μl well solution, and left overnight. Next day, the crystals were cryo-protected using well solution supplemented with 25% ethylene glycol and flash frozen in liquid nitrogen. The data were collected at the 22-ID beam line maintained by Southeast Regional Collaborative Access Team (SERCAT) at the Advanced Photon Source, Argonne National Laboratory. The data were indexed, integrated and scaled using the HKL2000 suite of programs^57^. Phase determination, structure refinement and model building were completed using Phaser, Refmac and Coot (part of the CCP4 package)^58^. The apo structure of PA_N_^ΔLoop^ (PDB ID: 5DES) was used as starting model for molecular replacement. The details of the data collection and refinement statistics are given in Table S1.

## Additional Information

**How to cite this article**: Carcelli, M. *et al*. N-acylhydrazone inhibitors of influenza virus PA endonuclease with versatile metal binding modes. *Sci. Rep.* **6**, 31500; doi: 10.1038/srep31500 (2016).

## Supplementary Material

Supplementary Information

## Figures and Tables

**Table 1:** Inhibitory activity of the N-acylhydrazones **1**–**27** and hydrazide **28** in the enzymatic assay with influenza virus PA-Nter endonuclease, or in cellular influenza virus assays.

Compound	Enzyme assay with PA-Nter^a^	Virus yield assay in influenza virus-infected MDCK cells^b^				vRNP reconstitution assay in HEK293T cells^c^	
		Antiviral activity		Cytotoxicity	SI^d^	Activity	Cytotoxicity
	IC_50_	EC_99_	EC_90_	CC_50_		EC_50_	CC_50_
**(1)**	24	ND^f^	ND	ND		107	>200
**(2)**	>500	ND	ND	ND		>100	>200
**(3)**	>500	ND	ND	>200		5.9	48
**(4)**	>500	ND	ND	>200		6.3	33
**(5)**	67	>25	>25	≥146		2.6	10
**(6)**	>500	>50	>50	>200		15	14
**(7)**	54	172	100	>200	>2.0	3.2	8.9
**(8)**	>500	>12.5	>12.5	>200		1.9	15
**(9)**	34	16	5.3	>200	>38	5.5	>200
**(10)**	68	14	8.5	111	>13	0.40	132
**(11)**	45	30	12	>200	>17	5.6	>200
**(12)**	>500	>12.5	>12.5	>200		20	39
**(13)**	69	71	34	>200	>5.9	6.3	>200
**(14)**	>500	63	37	>200	>5.4	2.3	>200
**(15)**	8.9	18	7.5	≥172	≥23	14	>200
**(16)**	454	67	28	>200	>7.1	5.2	>200
**(17)**	482	21	8.1	>200	>25	7.1	>200
**(18)**	83	6.2	2.2	>200	>91	3.3	>200
**(19)**	>500	53	26	>200	>7.7	5.7	>200
**(20)**	18	35	11	>200	>18	2.2	>200
**(21)**	13	8.3	3.6	>200	>56	2.5	>200
**(22)**	75	7.4	3.4	>200	>59	0.42	>200
**(23)**	8.7	11	3.5	>200	>57	3.1	>200
**(24)**	131	58	26	>200	>7.7	25	>200
**(25)**	40	132	70	>200	>2.9	4.1	>200
**(26)**	30	36	13	>200	>15	5.5	>200
**(27)**	36	ND	ND	ND		21	>200
**(28)**	40	158	85	>200	>2.4	7.2	>200
DPBA^e^	5.3	ND	ND	ND		ND	ND
Ribavirin	ND	13	8.5	>200	>24	9.4	>200

^a^Recombinant PA-Nter was incubated with the ssDNA plasmid substrate, a Mn^2+^-containing buffer and test compounds. Cleavage of the substrate was assessed after 2 hr incubation. The IC_50_ represents the compound concentration (in μM) required to obtain 50% inhibition of cleavage, calculated by nonlinear least-squares regression analysis (using GraphPad Prism software) of the results from 2–4 independent experiments.

^b^MDCK cells were infected with influenza A virus (strain A/PR/8/34) and incubated with the compounds during 24 h. The virus yield in the supernatant was assessed by real-time qPCR. The EC_99_ and EC_90_ values represent the compound concentrations (in μM) producing a 2-log_10_ or 1-log_10_ reduction in virus titer, respectively, determined in 2–3 independent experiments. The cytotoxicity, assessed in uninfected MDCK cells, was expressed as the CC_50_ value (50% cytotoxic concentration, determined with the MTS cell viability assay, in μM).

^c^HEK293T cells were co-transfected with the four vRNP-reconstituting plasmids and the luciferase reporter plasmid in the presence of the test compounds. The EC_50_ represents the compound concentration (in μM) producing 50% reduction in vRNP-driven firefly reporter signal, estimated at 24 h after transfection. The EC_50_ value was derived from data from 2–4 independent experiments, by nonlinear least-squares regression analysis (using GraphPad Prism software). The CC_50_ (in μM), i.e. the 50% cytotoxic concentration, was determined in untransfected HEK293T cells by MTS cell viability assay.

^d^SI, selectivity index, defined as the ratio between the CC_50_ and EC_90_.

^e^DPBA, 2,4-dioxo-4-phenylbutanoic acid.

^f^ND, not determined.
