# Supplementary material for: N-acylhydrazone inhibitors of influenza virus PA endonuclease with versatile metal binding modes
Source: Sci Rep. 2016 Aug 11;6:31500. doi: 10.1038/srep31500 (PMC4980666; doi:10.1038/srep31500)
Supplement: Supplementary Information [file srep31500-s1.pdf]

Supplementary Information

N-acylhydrazone inhibitors of influenza virus PA endonuclease with versatile metal binding modes

Mauro Carcelli,[*‡] Dominga Rogolino,[‡] Anna Gatti,[‡] Laura De Luca,[±] Mario Sechi,[†] Gyanendra Kumar,[§] Stephen W. White,[§] Annelies Stevaert,[♯] and Lieve Naesens[*♯]

[‡]Dipartimento di Chimica and Consorzio Interuniversitario di Ricerca in Chimica dei Metalli nei Sistemi Biologici, Università di Parma, Parco Area delle Scienze 17/A, 43124 Parma, Italy. [±]Dipartimento di Scienze del Farmaco e Prodotti per la Salute, Università di Messina, Polo Universitario SS. Annunziata, 98158 Messina, Italy. [†]Dipartimento di Chimica e Farmacia, Università di Sassari, Via Vienna 2, I-07100 Sassari, Italy. [♯]Rega Institute for Medical Research, KU Leuven – University of Leuven, B-3000 Leuven, Belgium. [§]Dept. of Structural Biology, St. Jude Children's Research Hospital, 262, Danny Thomas Place, Mail Stop 311, Memphis, TN 38105 U.S.A.

3,4,5-trihydroxybenzohydrazide (28). Light brown powder. Yield: 70%. ^{1}H-NMR (DMSO-d_6, 25°C), δ: 4.36 (s, br, 2H, NH_2), 6.79 (s, 2H, H_{arom}), 9.24 (s, br, 1H, NH); IR (cm^{-1}): ν_{NH} = 3424, 3390, 3296; $\nu_{C=O}$ = 1600.

3,4,5-trimethoxybenzohydrazide (29). White powder. Yield: 63%. ^{1}H-NMR (DMSO-d_6, 25°C), δ: 3.69 (s, 3H, OCH_3), 3.81 (s, 6H, OCH_3), 4.47 (s, br, 2H, NH_2), 7.16 (s, 2H, H_{arom}), 9.72 (s, br, 1H, NH); IR (cm^{-1}): ν_{NH} = 3455; $\nu_{C=O}$ = 1697; $\nu_{C=N}$ = 1596.

N'-(2,3-dihydroxybenzylidene)-semicarbazide (1). Yield = 54%. ^{1}H-NMR (DMSO-d_6, 25°C), δ: 6.36 (s, br, 2H, NH_2), 6.64 (t, J = 7.8 Hz, 1H, ArH), 6.76 (d, J = 7.6 Hz, 1H, ArH), 7.17 (d, 1H; J = 7.8, ArH), 8.14 (s, 1H; HC=N), 9.21 (s, br, 1H; OH), 9.39 (s, br, 1H; OH), 10.18 (s, br, 1H; NH). MS (EI, 70 eV) m/z (%) = 195.2 ($[M]^+$, 100); IR (cm^{-1}): ν_{NH} = 3455; ν_{OH} = 3166; $\nu_{C=O}$ = 1694; $\nu_{C=N}$ = 1592. Anal. Calcd for $C_8H_9N_3O_3$: C 49.23; H 4.65; N 21.53. Found: C 49.64; H 4.71; N 21.71.

N'-(2-hydroxy-3-methoxybenzylidene)-semicarbazide (2). Yield = 81%. ^{1}H-NMR (DMSO-d_6, 25°C), δ: 3.84 (s, 3H, OCH3), 6.39 (s, br, 2H, NH_2), 6.76 (t, J = 7.8 Hz, 1H, ArH), 6.92 (d, J = 7.6 Hz, 1H, ArH), 7.37 (d, 1H; J = 7.8, ArH), 8.17 (s, 1H; HC=N), 9.20 (s, br, 1H; OH), 10.20 (s, br, 1H; NH). MS (EI, 70 eV) m/z (%) = 209.2 ($[M]^+$, 100); IR (cm^{-1}): ν_{NH} = 3466; ν_{OH} = 3170-3280 (br); $\nu_{C=O}$ = 1672; $\nu_{C=N}$ = 1586. Anal. Calcd for $C_9H_{11}N_3O_3{\cdot}1/4H_2O$: C 50.58; H 5.42; N 19.66. Found: C 50.67; H 5.37; N 19.44.

N'-(2,3-dihydroxybenzylidene)heptylhydrazide (3). Yield = 95%. ^{1}H-NMR (DMSO-d_6, 25°C), δ: 0.83-0.86 (m, CH_3); 1.16-1.26 (m, CH_2); 1.54-1.59 (m, CH_2); 2.17-2.29 (m, CH_2); 2.53-2.59 (m, 6.64-6.73), (m, overlapping isomers, ArH), 6.81-6.84 (m, overlapping isomers, ArH), 6.92 (d, isomer E, ArH), 7.03 (d, isomer Z, ArH), 8.23 (s, HC=N, isomer E), 8.28 (s, HC=N, isomer Z), 9.21 (s, br, OH), 9.49 (s, br, OH), 11.06, 11.20, 11.59 (s, br, NH+OH). MS (EI, 70 eV) m/z (%) = 301 ($[M+Na]^+$, 100); IR (cm^{-1}): ν_{NH} = 3490; ν_{OH} = 2922-2941; $\nu_{C=O}$ = 1663. Anal. Calcd for $C_{15}H_{22}N_2O_3{\cdot}1/2H_2O$: C 62.70; H 8.07; N 9.75. Found: C 62.84; H 7.97; N 9.88.

N'-(2-hydroxy-3-methoxybenzylidene)heptylhydrazide (4). Yield = 41%. ^{1}H-NMR (DMSO-d_6, 25°C), δ (overlapping isomers): 0.83-0.86 (m, CH_3); 1.26-1.27 (m, CH_2); 1.55-1.59 (m, CH_2); 2.18-

2.23 (m, CH_2); 2.52-2.57 (m, CH_2), 3.79 (s, br, OCH_3), 6.80-6.86 (m, ArH), 6.94-7.01 (m, ArH), 7.08 (d, isomer E, ArH), 7.20 (d, isomer Z, ArH), 8.27 (s, HC=N, isomer E), 8.34 (s, HC=N, isomer Z), 9.54 (s, br, OH), 10.96, 11.25, 11.57 (s, br, NH+OH). MS (EI, 70 eV) m/z (%) = 292.2 ($[M]^+$, 24); IR (cm^{-1}): ν_{NH} = 3182; ν_{OH} = 3072, 2917; $\nu_{C=O}$ = 1663. Anal. Calcd for $C_{16}H_{24}N_2O_3$: C 65.73; H 8.27; N 9.58. Found: C 65.76; H 8.42; N 9.45.

N'-(2,3-dihydroxybenzylidene)benzoylhydrazide (5). Yield = 32%. ^{1}H-NMR (DMSO-d_6, 25°C), δ: 6.74 (t, J = 7.8 Hz, 1H, ArH), 6.86 (d, J = 7.2 Hz, 1H, ArH), 6.97 (d, J = 7.2 Hz, 1H, ArH), 7.52-7.61 (m, 3H; ArH), 7.94 (d, 2H; J = 7.2, ArH), 8.59 (s, 1H; HC=N), 9.28 (s, br, 1H; OH), 11.16 (s, br, 1H; NH), 12.14 (s, br, 1H; OH). MS (EI, 70 eV) m/z (%) = 256.2 ($[M]^+$, 100); IR (cm^{-1}): ν_{NH} = 3280; ν_{OH} = 3047; $\nu_{C=O}$ = 1658; $\nu_{C=N}$ = 1527. Anal. Calcd for $C_{14}H_{12}N_2O_3 \cdot 1/4H_2O$: C 64.48; H 4.83; N 10.74. Found: C 64.64; H 4.71; N 10.71.

N'-(2-hydroxy-3-methoxybenzylidene)benzoylhydrazide (6). Yield = 89%. ^{1}H-NMR (DMSO-d_6, 25°C), δ: 3.81 (s, 3H, OCH_3), 6.87 (t, J = 7.8 Hz, 1H, ArH), 7.04 (d, J = 7.2 Hz, 1H, ArH), 7.15 (d, J = 7.2 Hz, 1H, ArH), 7.52-7.64 (m, 3H; ArH), 7.94 (d, 2H; J = 6.9, ArH), 8.65 (s, 1H; HC=N), 11.01 (s, br, 1H; NH), 12.05 (s, br, 1H; OH). MS (EI, 70 eV) m/z (%) =270.1 ($[M]^+$, 100); IR (cm^{-1}): ν_{NH+OH} = 2830-3072 (br); $\nu_{C=O}$ = 1657; ν_{OCH3} = 1249, 1076. Anal. Calcd for $C_{15}H_{14}N_2O_3 \cdot H_2O$: C 62.49; H 5.59; N 9.72. Found: C 62.88; H 5.59; N 9.94.

N'-(2,3-dihydroxybenzylidene)-2-hydroxybenzoylhydrazide (7). Yield = 30%. ^{1}H-NMR (DMSO-d_6, 25°C), δ: 6.75 (t, J = 7.8 Hz, 1H, ArH), 6.87 (d, J = 7.2 Hz, 1H, ArH), 6.94-7.00 (m, 3H, ArH), 7.43 (t, J = 7.8 Hz, 1H; ArH), 7.88 (d, 2H; J = 7.2, ArH), 8.64 (s, 1H; HC=N), 9.32 (s, br, 1H; OH), 11.03 (s, br, 1H; NH), 12.05 (s, br, 2H; OH). MS (EI, 70 eV) m/z (%) = 272.1 ($[M]^+$, 100); IR (cm^{-1}): ν_{NH+OH} = 2980-3256 (br); $\nu_{C=O}$ = 1635. Anal. Calcd for $C_{14}H_{12}N_2O_4$·: C 61.76; H 4.44; N 10.29. Found: C 61.97; H 4.43; N 10.20.

N'-(2-hydroxy-3-methoxybenzylidene)-2-hydroxybenzoylhydrazide (8). Yield = 89%. ^{1}H-NMR (DMSO-d_6, 25°C), δ: 3.82 (s, 3H, OCH_3), 6.85-7.06 (m, 4H, ArH), 7.17 (d, J = 7.5 Hz, 1H, ArH), 7.45 (t, J = 7.6 Hz, 1H; ArH), 7.89 (d, 2H; J = 7.5, ArH), 8.69 (s, 1H; HC=N), 10.87 (s, br, 1H;

NH), 11.99 (s, br, 2H; OH). MS (EI, 70 eV) m/z (%) = 286.0 ($[M]^+$, 100); IR (cm^{-1}): ν_{NH+OH} = 2993-3211 (br); $\nu_{C=O}$ = 1606; $\nu_{C=N}$ = 1560; ν_{OCH3} = 1256, 1079. Anal. Calcd for $C_{15}H_{14}N_2O_4 \cdot 1/2H_2O$: C 61.01; H 5.12; N 9.49. Found: C 61.20; H 4.89; N 9.58.

N'-(2,4,5-trihydroxyphenyl)-2-hydroxybenzoylhydrazide (9) Yield = 84%. ^{1}H-NMR (DMSO-d_6, 25°C), δ: 6.35 (s, 1H, ArH), 6.93-6.98 (m, 3H, ArH), 7.44 (t, J = 6.9, 1H, ArH), 7.89 (d, J = 7.0, 1H, ArH), 8.50 (s, 1H; HC=N), 8.62 (s, br, 1H; OH), 9.61 (s, br, 1H; OH), 10.50 (s, br, 1H, NH), 11.81, 12.00 (s, br, 1H; OH). MS (EI, 70 eV), m/z (%) =288.1 ($[M]^+$, 20); IR (cm^{-1}): ν_{NH+OH} = 3274-3420 (br); $\nu_{C=O}$ = 1634. Anal. Calcd for $C_{14}H_{12}N_2O_5 \cdot H_2O$: C 54.90; H 4.61; N 9.15. Found: C 54.65; H 4.72; N 9.29.

N'-(2,4,6-trihydroxyphenyl)-2-hydroxybenzoylhydrazide (10) Yield = 65%. ^{1}H-NMR (DMSO-d_6, 25°C), δ: 5.86 (s, 2H, ArH), 6.93-6.98 (m, 2H, ArH), 7.45 (t, J = 6.9, 1H, ArH), 7.88 (d, J = 7.0, 1H, ArH), 8.83 (s, 1H; HC=N), 9.89 (s, br, 1H; OH), 11.09 (s, br, 1H; NH), 11.95, 11.97 (s, br, 1H; OH). MS (EI, 70 eV), m/z (%) =288.1 ($[M]^+$, 20); IR (cm^{-1}): ν_{NH+OH} = 3100-3360 (br); $\nu_{C=O}$ = 1631. Anal. Calcd for $C_{14}H_{12}N_2O_5 \cdot H_2O$: C 54.90; H 4.61; N 9.15. Found: C 54.82; H 4.52; N 9.42.

N'-(3,4,5-trihydroxyphenyl)-2-hydroxybenzoylhydrazide (11) Yield = 76 %. ^{1}H-NMR (DMSO-d_6, 25°C), δ: 6.72 (s, 2H, ArH), 6.92-6.97 (m, 2H, ArH), 7.43 (t, J = 7.1, 1H, ArH), 7.88 (d, J = 7.0, 1H, ArH), 8.19 (s, 1H; HC=N), 8.67 (s, br, 1H; OH), 9.18 (s, br, 2H; OH), 11.67 (s, br, 2H; NH), 12.03 (s, br, 1H; OH). MS (EI, 70 eV), m/z (%) =287.9 ($[M]^+$, 35); IR (cm^{-1}): ν_{NH+OH} = 3280-3321 (br); $\nu_{C=O}$ = 1637. Anal. Calcd for $C_{14}H_{12}N_2O_5$: C 58.33; H 4.20; N 9.72. Found: C 58.19; H 4.03; N 9.54.

N'-(2,5-dihydroxybenzylidene)-3,4,5-trimethoxybenzoylhydrazone (12). Yield = 90%. ^{1}H-NMR (DMSO-d_6, 25°C), δ: 3.74 (s, 3H, OCH3), 3.87 (s, 6H, OCH3), 6.74 (m, 2H, ArH), 7.00 (s, 1H, ArH), 7.27 (s, 2H, ArH), 8.59 (s, 1H; HC=N), 8.98 (br, 1H; OH), 10.34 (s, br, 1H; NH), 11.83 (s, br, 1H; OH). MS (EI, 70 eV), m/z (%) = 346.3 ($[M]^+$, 100); IR (cm^{-1}): ν_{NH} = 3274, ν_{OH} = 3090-3160 (br); $\nu_{C=O}$ = 1654. Anal. Calcd for $C_{17}H_{18}N_2O_6$: C 58.96; H 5.24; N 8.09. Found: C 58.82; H 5.52; N 8.15.

N'-phenyl-3,4,5-trihydroxybenzoylhydrazide (13) Yield = 70%. ^{1}H-NMR (DMSO-d$_6$, 25°C), δ: 6.93 (s, 2H; ArH), 7.44 (m, 3H, ArH), 7.68 (d, J = 7.9 Hz, 2H, ArH), 8.41 (s, 1H; HC=N), 8.84 (s, br, 1H; OH), 9.15 (s, br, 2H; OH), 11.54 (s, br, 1H; NH). MS (EI, 70 eV), m/z (%) = 272.1 ([M]$^+$, 20); IR (cm^{-1}): ν_{OH} = 3534, ν_{NH+OH} = 3226-3327, (br); $\nu_{C=O}$ = 1590. Anal. Calcd for $C_{14}H_{12}N_2O_4$.: C 61.76; H 4.44; N 10.29. Found: C 61.55; H 4.67; N 10.04.

N'-(2-hydroxy-benzylidene)-3,4,5-trihydroxybenzoylhydrazone (14). Yield = 79%. ^{1}H-NMR (DMSO-d$_6$, 25°C), δ: 6.90-6.95 (m, 4H; ArH), 7.29 (t, 1H, J = 7.9 Hz, ArH), 7.47 (d, J = 7.8 Hz, 2H, ArH), 8.58 (s, 1H; HC=N), 8.89 (s, 1H; OH), 9.20 (s, br, 2H; OH), 11.49 (s, 1H; OH), 11.84 (s, br, 1H; NH). MS (EI, 70 eV), m/z (%) = 288.1 ([M]$^+$, 25); IR (cm^{-1}): ν_{OH} = 3534, ν_{NH+OH} = 3226-3327, (br); $\nu_{C=O}$ = 1590. Anal. Calcd for $C_{14}H_{12}N_2O_5$.: C 58.33; H 4.20; N 9.72. Found: C 58.67; H 4.25; N 9.80.

N'-(2,5-dihydroxybenzylidene)-3,4,5-trihydroxybenzoylhydrazide (15). Yield = 59%. ^{1}H-NMR (DMSO-d$_6$, 25°C), δ: 6.74 (m, 2H; ArH), 6.89 (s, 1H, ArH), 6.94 (s, 2H, ArH), 8.50 (s, 1H; HC=N), 8.89, 9.00 (br, 2H; OH), 9.22 (s, br, 1H; OH), 10.60 (s, br, 1H; NH), 11.74 (s, br, 1H; OH). MS (EI, 70 eV), m/z (%) = 304.1 ([M]$^+$, 100); IR (cm^{-1}): ν_{OH} = 3403 (br); ν_{NH+OH} = 3215 (br); $\nu_{C=O}$ = 1592. Anal. Calcd for $C_{14}H_{12}N_2O_6 \cdot H_2O$: C 52.18; H 4.38; N 8.69. Found: C 52.44; H 4.44; N 8.58.

N'-(2-hydroxy-5-methoxybenzylidene)-3,4,5-trihydroxybenzoylhydrazide (16) Yield = 92%. ^{1}H-NMR (DMSO-d$_6$, 25°C), δ: 3.73 (s, 3H, OCH_3), 6.84-6.91 (m, 2H, ArH), 6.94 (s, 1H, ArH), 7.06 (s, 1H, ArH), 8.56 (s, 1H; HC=N), 8.97 (br, 3H; OH), 10.89 (s, br, 1H; NH), 11.83 (s, br, 1H; OH). MS (EI, 70 eV), m/z (%) = 318.3 ([M]$^+$, 100); IR (cm^{-1}): ν_{OH} = 3496, ν_{NH+OH} = 3178 (br); $\nu_{C=O}$ = 1654. Anal. Calcd for $C_{15}H_{14}N_2O_6$: C 56.60; H 4.43; N 8.80. Found: C 56.89; H 4.32; N 8.96.

N'-(2,4-dihydroxybenzylidene)-3,4,5-trihydroxybenzoylhydrazide (17). Yield = 43%. ^{1}H-NMR (DMSO-d$_6$, 25°C), δ: 6.31 (s, 1H; ArH), 6.36 (dd, J = 7.9 Hz, 1H, ArH), 6.92 (s, 2H, ArH), 7.24 (d, J = 7.8 Hz, 1H, ArH), 8.45 (s, 1H; HC=N), 9.18 (s, br, 2H; OH), 9.93 (s, br, 1H; OH), 11.63 (m, br, 2H; NH+OH). MS (EI, 70 eV), m/z (%) = 304.1 ([M]$^+$, 100; IR (cm^{-1}): ν_{OH} = 3552; ν_{NH+OH} = 3261-

3320 (br); $\nu_{C=O}$ = 1630; $\nu_{C=N}$ = 1565. Anal. Calcd for $C_{14}H_{12}N_2O_6$.: C 55.27; H 3.98; N 9.21. Found: C 55.02; H 3.88; N 9.20.

N'-(2,3-dihydroxybenzylidene)-3,4,5-trihydroxybenzoylhydrazide (18). Yield = 83%. ^{1}H-NMR (DMSO-d$_6$, 25°C), δ: 6.73 (t, J = 7.8 Hz, 1H, ArH), 6.84 (d, J = 7.2 Hz, 1H, ArH), 6.90 (d, 1H, ArH), 6.95 (s, 2H; ArH), 8.53 (s, 1H; HC=N), 8.89 (s, br, 1H; OH), 9.12 (s, br, 1H; OH), 9.21 (s, br, 2H; OH), 11.41 (s, br, 1H; NH), 11.83 (s, br, 1H; OH). MS (EI, 70 eV) m/z (%) = 304.0 ([M]$^+$, 100);): ν_{NH+OH} = 3255 (br); $\nu_{C=O}$ = 1654. Anal. Calcd for $C_{14}H_{12}N_2O_6$.: C 55.27; H 3.98; N 9.21. Found: C 55.04; H 4.12; N 9.15.

N'-(2-hydroxy-3-methoxybenzylidene)-3,4,5-trihydroxybenzoylhydrazide (19, H_2L). Yield = 61%. ^{1}H-NMR (DMSO-d$_6$, 25°C), δ: 3.81 (s, 3H, OCH_3), 6.85 (t, J = 7.9 Hz, 1H, ArH), 6.94 (s, 2H; ArH), 7.03 (d, J = 8.1 Hz, 1H, ArH), 7.08 (d, 1H, J = 7.9 Hz, ArH), 8.58 (s, 1H; HC=N), 8.90 (s, br, 1H; OH), 9.19 (s, br, 2H; OH), 11.25 (s, br, 1H; NH), 11.80 (s, br, 2H; OH). ^{1}H-NMR (MeOD-d$_4$, 25°C), δ: 3.91 (s, 3H, OCH_3), 6.91 (t, J = 7.9 Hz, 1H, ArH), 7.03-7.07 (m, 3H; ArH), 7.23 (d, J = 7.6 Hz, 1H, ArH), 8.57 (s, 1H; HC=N). ^{13}C-NMR (MeOD-d$_4$, 25°C), δ: 55.43; 107.11; 113.72; 118.87; 119.10; 121.39; 122.90; 137.54; 145.61; 147.48; 148.17; 148.54; 164.75. MS (EI, 70 eV) m/z (%) = 318.0 ([M]$^+$, 100); IR (cm^{-1}): ν_{OH} = 3418; ν_{NH+OH} = 3222 (br); $\nu_{C=O}$ = 1664; $\nu_{C=N}$ = 1598; ν_{OCH3} = 1252, 1033. Anal. Calcd for $C_{15}H_{14}N_2O_6 \cdot H_2O$: C 53.57; H 4.80; N 8.33. Found: C 53.50; H 4.88; N 7.92.

N'-(2,3,4-trihydroxyphenyl)-3,4,5-trihydroxybenzoylhydrazide (20). Yield = 25%. ^{1}H-NMR (DMSO-d$_6$, 25°C), δ: 6.38 (d, J = 9 Hz, 1H, ArH), 6.72 (d, J = 8.9 Hz, 1H, ArH), 6.92 (s, 2H, ArH), 8.41 (s, 1H; HC=N), 8.44 (s, br, 1H; OH), 8.86 (s, br, 1H; OH), 9.19 (s, br, 2H; OH), 9.40 (s, br, 1H; OH), 11.66 (s, br, 1H; NH), 11.72 (s, br, 1H; OH). MS (EI, 70 eV), m/z (%) = 319.9 ([M]$^+$, 40); IR (cm^{-1}): ν_{NH+OH} = 3298-3420 (br); $\nu_{C=O}$ = 1608. Anal. Calcd for $C_{14}H_{12}N_2O_7$.: C 52.51; H 3.78; N 8.75. Found: C 52.42; H 3.88; N 8.56.

N'-(2,4,5-trihydroxyphenyl)-3,4,5-trihydroxybenzoylhydrazide (21). Yield = 83%. ^{1}H-NMR (DMSO-d$_6$, 25°C), δ: 6.32 (s, 1H, ArH), 6.79 (s, 1H, ArH), 6.91 (s, 2H, ArH), 8.38 (s, 1H; HC=N),

8.53 (s, br, 1H; OH), 8.83 (s, br, 1H; OH), 9.16(s, br, 2H; OH), 9.52 (s, br, 1H; OH), 10.83 (s, br, 1H, NH), 11.52 (s, br, 1H; OH). MS (EI, 70 eV), m/z (%) = 320.1 ($[M]^+$, 20); IR (cm^{-1}): ν_{NH+OH} = 3250-3300 (br); $\nu_{C=O}$ = 1635. Anal. Calcd for $C_{14}H_{12}N_2O_7$.: C 52.51; H 3.78; N 8.75. Found: C 52.35; H 4.02; N 8.67.

N'-(2,4,6-trihydroxyphenyl)-3,4,5-trihydroxybenzoylhydrazide (22). Yield = 72%. ^{1}H-NMR (DMSO-d_6, 25°C), δ: 5.83 (s, 2H, ArH), 6.90 (s, 2H, ArH), 8.75 (s, 1H; HC=N), 8.85 (s, br, 1H; OH), 9.18 (s, br, 2H; OH), 9.77 (s, br, 1H; OH), 11.12 (s, br, 2H; NH+OH), 11.60 (s, br, 1H; OH). MS (EI, 70 eV), m/z (%) = 320.2 ($[M]^+$, 35); IR (cm^{-1}): ν_{NH+OH} = 3298-3420 (br); $\nu_{C=O}$ = 1608. Anal. Calcd for $C_{14}H_{12}N_2O_7$.: C 52.51; H 3.78; N 8.75. Found: C 52.74; H 3.51; N 8.53.

N'-(3,4,5-trihydroxyphenyl)-3,4,5-trihydroxybenzoylhydrazide (23). Yield = 73%. ^{1}H-NMR (DMSO-d_6, 25°C), δ: 6.65 (s, 2H, ArH), 6.89 (s, 2H, ArH), 8.12 (s, 1H; HC=N), 8.55 (s, br, 1H; OH), 8.80 (s, br, 1H; OH), 9.11-9.14 (m, br, 4H; NH+OH), 11.26 (s, br, 1H; OH). MS (EI, 70 eV), m/z (%) = 320.2 ($[M]^+$, 35); IR (cm^{-1}): ν_{NH+OH} = 3244-3343 (br); $\nu_{C=O}$ = 1620. Anal. Calcd for $C_{14}H_{12}N_2O_7$.: C 52.51; H 3.78; N 8.75. Found: C 52.55; H 3.92; N 8.73.

N'-(2-pyridyl)-3,4,5-trihydroxybenzoylhydrazide (24). Yield = 55%. ^{1}H-NMR (DMSO-d_6, 25°C), δ: 6.97 (s, 2H; ArH), 7.67 (m, 1H, ArH), 8.12-8.22 (m, 2H, ArH), 8.58 (s, 1H; HC=N), 8.71 (d, 1H; ArH). MS (EI, 70 eV), m/z (%) = 273.1 ($[M]^+$, 20); IR (cm^{-1}): ν_{OH} = 3531, ν_{NH+OH} = 3216-3297, (br); $\nu_{C=O}$ = 1598. Anal. Calcd for $C_{13}H_{11}N_3O_4$.: C 57.14; H 4.06; N 15.38. Found: C 57.35; H 4.10; N 15.43

N'-(2-pyrrolidyl)-3,4,5-trihydroxybenzoylhydrazide (25). Yield = 25%. ^{1}H-NMR (DMSO-d_6, 25°C), δ: 6.12 (s, 1H; ArH), 6.43 (s, 1H, ArH), 6.89 (s, 3H, ArH), 8.24 (s, 1H; HC=N), 8.75, 9.10 (s, br, 3H; OH), 11.20 (s, br, 1H; NH), 11.44 (s, br, 1H; OH). MS (EI, 70 eV), m/z (%) = 261.0 ($[M]^+$, 15); IR (cm^{-1}): ν_{NH+OH} = 3215 (br); $\nu_{C=O}$ = 1600. Anal. Calcd for $C_{12}H_{11}N_3O_4$.: C 55.17; H 4.24; N 16.09. Found: C 55.22; H 4.43; N 15.79.

N'-(3-(1,1'-biphenyl)-4-carboxylic acid)-3,4,5-trihydroxybenzoylhydrazide (26). Yield = 51%. ^{1}H-NMR (DMSO-d_6, 25°C), δ: 6.95 (s, 2H; ArH), 7.59 (t, J = 7.6 Hz, 1H, ArH), 7.77 (m, 2H, ArH),

7.86 (d, 2H, J = 7.8 Hz, ArH), 8.02 (s, 1H, ArH), 8.07 (d, 2H, J = 7.7 Hz, ArH), 8.50 (s, 1H; HC=N), 8.86 (s, br, 1H, OH), 9.18 (s, br, 2H; OH), 11.65 (s, br, 1H; NH), 12.98 (s, br, 1H; COOH). MS-ESI, m/z (%) = 415 ($[M+Na]^+$, 80); IR (cm^{-1}): $\nu_{C=O}$ = 1676 (br), $\nu_{C=N}$ = 1599. Anal. Calcd for $C_{21}H_{16}N_2O_6 \cdot 2.5H_2O$: C 57.67; H 4.84; N 6.40. Found: C 57.11; H 4.84; N 6.40.

N'-(3,4,5-trihydroxybenzylidene)-semicarbazide (27). Yield = 62%. ^{1}H-NMR (DMSO-d_6, 25°C), δ: 6.27 (s, br, 2H, NH_2), 6.81 (s, 2H, ArH), 7.61 (s, 1H; HC=N), 8.57-8.96 (br, 3H; OH), 9.98 (s, br, 1H; NH). MS (EI, 70 eV) m/z (%) = 211.1 ($[M]^+$, 60); IR (cm^{-1}): ν_{NH} = 3460; ν_{OH} = 3170-3186; $\nu_{C=O}$ = 1690; $\nu_{C=N}$ = 1594. Anal. Calcd for $C_8H_9N_3O_4$: C 45.50; H 4.30; N 19.90. Found: C 45.62; H 4.41; N 20.05.

$Mg(HL)_2 \cdot 4H_2O$. Method A. 100 mg (0.37 mmol) of **H_2L** were dissolved in 30 ml of methanol and 1 eq. of NEt_3 was added. The solution turned yellow and it was stirred at r.t. for 30 minutes. Upon addition of 0.5 eq. of $Mg(CH_3COO)_2 \cdot 4H_2O$ (40 mg), a light yellow precipitate was formed, which was stirred at r.t. for 3 hours; the reaction mixture was cooled overnight and then the precipitate was filtered off and washed with water. Method B. The same as method A with 0.37 mmol of ligand and 1 equivalent of $Mg(CH_3COO)_2 \cdot 4H_2O$. Method C. The same as method A with 4 equivalents of base. Light yellow powder. Yield: 58%. ^{1}H-NMR (MeOD, 25°C), δ: 3.89 (s, 3H, OCH_3); 6.89 (t, *J* = 7.8 Hz, 1H, ArH); 6.99-7.04 (m, 3H, ArH); 7.13 (d, *J* = 7.6 Hz, 1H); 8.51 (s, 1H; HC=N). ^{13}C-NMR (MeOD-d_4, 25°C), δ: 55.26; 106.89; 113.60; 118.69; 118.96; 121.35; 122.70; 137.56; 145.46; 147.44; 148.07; 148.65; 165.26. ^{1}H-NMR (DMSO-d_6, 25°C), δ: 3.89 (s, OCH_3); 3.85 (s, OCH_3, decoordinated ligand); 6.85-6.87 (overlapping signals); 6.96-7.07 (overlapping signals); 7.13-7.15 (overlapping signals); 8.51 (s, HC=N), 8.60 (s, HC=N, decoordinated ligand). MS-ESI, m/z (%) = 659 ($[M+H]^+$, 60); IR (cm^{-1}): ν_{NH+OH} = 3476, 3322 (br); $\nu_{C=O}$ = 1643. Anal. Calcd for $C_{30}H_{26}N_4O_{12}Mg \cdot 4H_2O$: C 49.30; H 4.69; N 7.67. Found: C 49.54; H 4.87; N 7.59.

Figure S1. UV-visible titration of ligand **23** (**A**) and **19** (**B**) with increasing amount of $Mg(CH_3COO)_2$

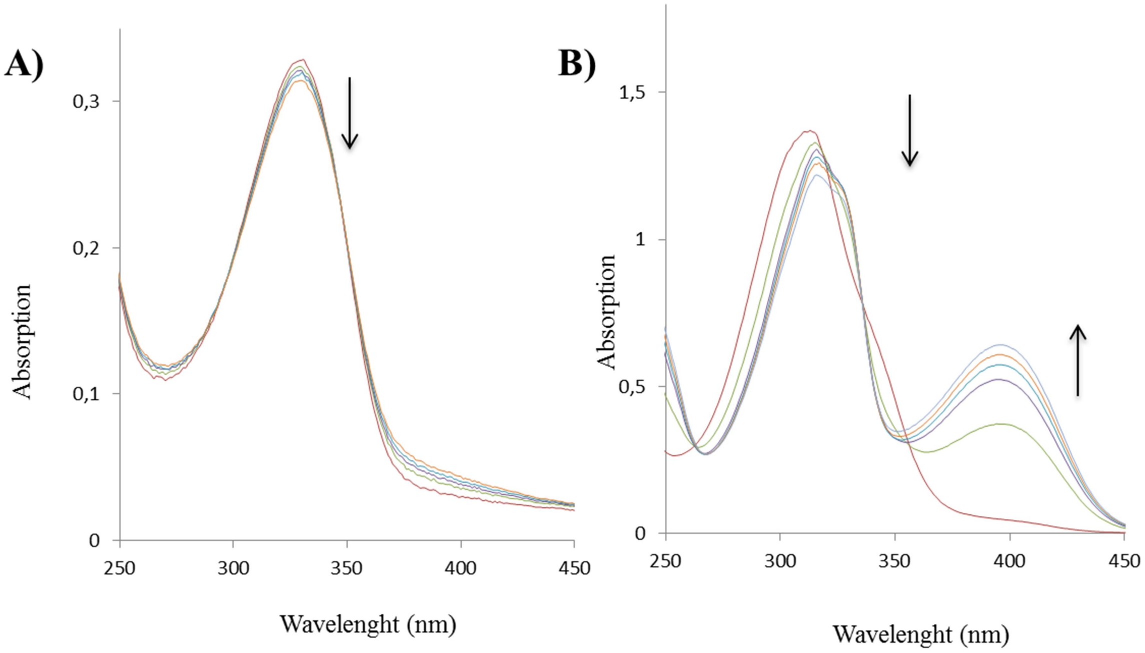

Figure S2. Dose-response curves for the biological activity of compounds **10**, **13** and **23**. Left panels: enzymatic assay with PA-Nter; right panels: cell-based vRNP reconstitution assay. The graphs were made by non-linear regression analysis on data from 2 to 4 experiments using GraphPad Prism

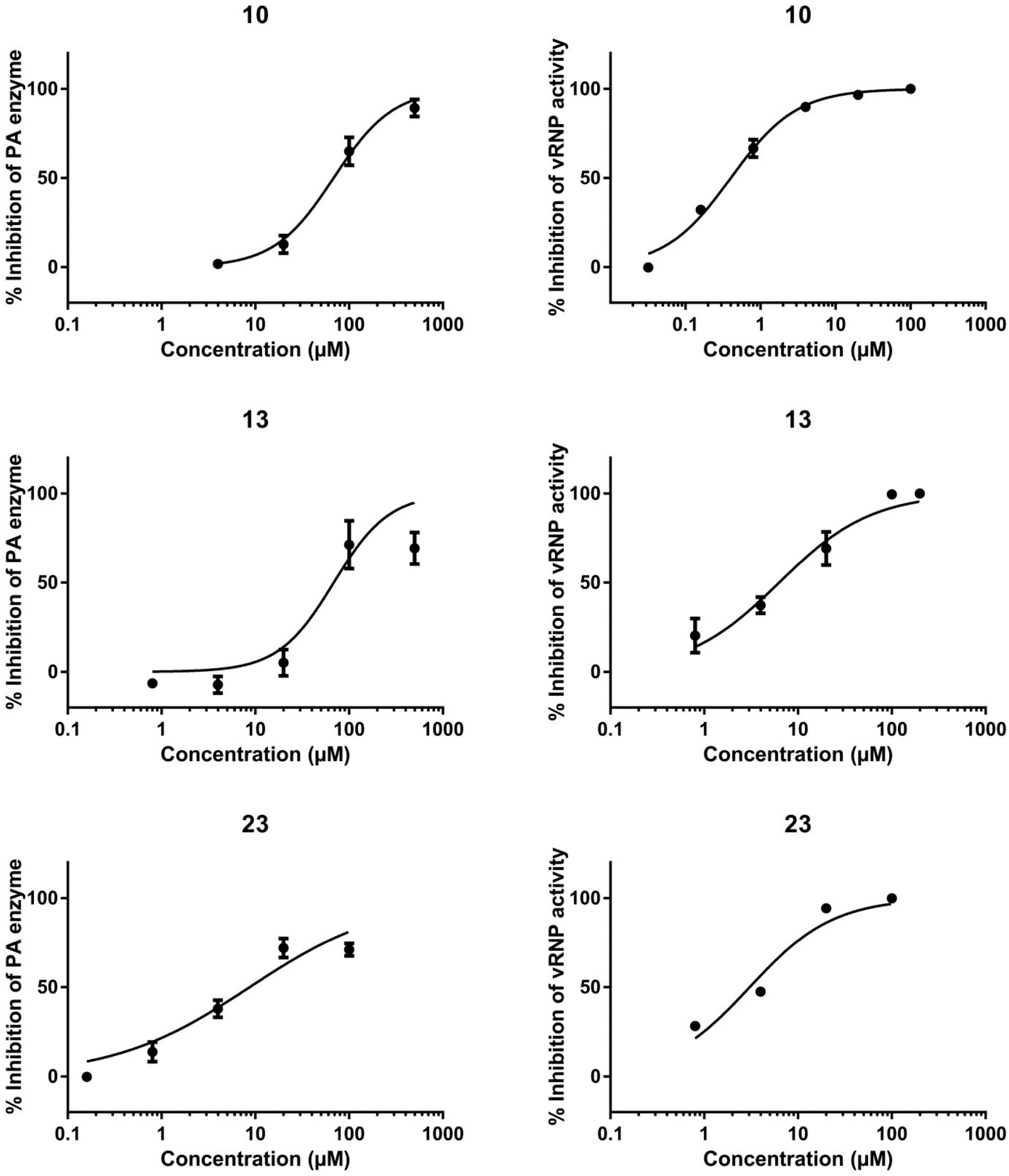

Table S1: Crystallographic data collection and refinement statistics for the $PA_N^{\Delta Loop}$-Compound **23** complex.

**PDB code**	5EGA
**Data collection**	
Space group	$P6_422$
a, b, c (Å)	73.99, 73.99, 128.55
α, β, γ (°)	90.0, 90.0, 120.0
Resolution (Å)*	50.0-2.15 (2.23-2.15)
R_{meas}*	7.3 (96.2)
*I/σI**	42.8 (3.0)
Completeness (%)	99.4
Redundancy	16.7
Wilson B-factor ($Å^2$)	54.5
**Refinement**	
Resolution*	50.0-2.15 (2.21-2.15)
No. of reflections	11252
R_{work}/ R_{free}	20.7/23.4
No. of atoms	
Protein	1405
Mn	2
Ligand	23
Water	32
B-factor ($Å^2$)	
Protein	64.11
Metal	57.25
Ligand	89.26
Water	56.31
r.m.s. deviations	
Bond lengths (Å)	0.012
Bond angles (Å)	1.415
Ramachandran Plot	
% residues in favoured region	98.3
% residues in allowed region	1.7
% residues in outlier region	0

*Highest resolution shell is shown in parenthesis.
